# Synthesis of a New Imidazole Amino Acid Ionic Liquid Polymer and Selective Adsorption Performance for Tea Polyphenols

**DOI:** 10.3390/polym12102171

**Published:** 2020-09-23

**Authors:** Yingjie Luo, Xiaoxia Huang, Shun Yao, Lincai Peng, Fulin Li, Hang Song

**Affiliations:** Department of Pharmaceutical and Biological Engineering, School of Chemical Engineering, Sichuan University, Chengdu 610000, China; luoyingjie1115@163.com (Y.L.); 15528091852@163.com (X.H.); cusack@scu.edu.cn (S.Y.); wacord@163.com (L.P.); lifulin2017@163.com (F.L.)

**Keywords:** ionic liquid polymer, imidazole, amino acid, adsorption, tea polyphenols

## Abstract

A series of imidazolium ionic liquid monomers with L-Proline anions (ViImCn-L-Pro and (ViIm)2Cn(L-Pro)2) were firstly synthesized, after which new copolymer materials were prepared by polymerization of the ionic liquid monomers with N,N′-methylene diacrylamide (MBA). Polymerization conditions, including the ratio of Ils(ViImC_n_-L-Pro or (ViIm)_2_C_n_(L-Pro)_2_) and MBA, solvent, ionic liquids and initiator’s amount, were investigated and found to have an important effect on the adsorption capacity. Polymerization conditions were shown to have more significant impacts on adsorption capacities in the following order: the ratio of Ils and MBA > the amount of initiator > ionic liquids > solvent. The polymers were characterized by IR, EA, SEM, particle size distribution and TG. One of the polymers exhibited the highest selective adsorption capacity of tea polyphenols (521 mg/g). which was significantly higher than other adsorption media. The absorbed tea polyphenols could be desorbed readily with 2% hydrochloric acid methanol solution as eluent. The polymer material could maintain a higher adsorption capacity after four reuses. Based on this polymer, a new method for the efficient separation of tea polyphenols from tea water could be developed.

## 1. Introduction

Ionic liquids (ILs) are a developing material and have been widely applied in extraction, adsorption and catalysis. Some dicationic ILs exhibit, in their structures, a higher adsorption capacity [1,2]. Poly(ionic liquid)s combine the characteristic of ILs with polymers and have many excellent properties, such as a better mechanical stability, ionic conductivity, processability, durability, chemical compatibility and controllability [3,4]. Poly(ionic liquid)s have special structures and could therefore have multiple functions [5,6,7,8]. In recent years, polyionic liquids have attracted great attention in the fields of materials, separation and purification, electrochemistry and catalysts [9,10,11,12]. The safety of imidazole-based ILs has been demonstrated in the past years [13]. Some natural amino acid-based imidazole ILs have a good biocompatibility, nontoxicity and other characteristics, and thus a better safety [14,15,16,17,18]. 

Tea polyphenols (TPs), including Epigallocatechin gallate (EGCG), Epicatechin (EC) and Epigallocatechin gallate (ECG), constitute a high content in tea, accounting for 15–30% of the dry weight of tea. TPs have multiple biological activities, such as oxidation resistance, preventing cardiovascular diseases or relieving atherosclerosis and arrhythmia [19,20]. TPs are difficult to obtain by chemical synthesis due to their complex chemical structures and poor chemical stability. Therefore, TP product is made mainly from tea, and its production involves a complex process that is composed of a series of processes. The TPs and other substances are first extracted from tea raw materials and then purified. To obtain high-purity TPs, a series of different separation methods and equipment have to be applied to remove various other compounds, such as pectin, polysaccharide, theophylline, caffeine and pigment [21].

Resin adsorption is one of purification methods used for TPs. For example, Dong and others [22] chemically synthesized adsorption resin NKA-2 as an adsorbent, and 80% ethanol was used as effluent solvent. However, the investment and operation costs are relatively high. In particular, the adsorption process has to use a large amount of volatile organic solvents, so the effects on ecological environmental protection and safety are not satisfactory. In recent years, several new methods for the separation and purification of TPs have been reported. Luo and others [23] prepared a porous starch that exhibited a selective adsorption performance on TPs with an adsorption capacity of 25.72 mg/g. It has some advantages, such as less organic solvent, mild adsorption conditions and good adsorption selectivity. However, a low adsorption capacity would lead to very large amount of adsorbent being required, which is difficult to realize in industrial applications. A good adsorption selectivity and high adsorption capacity are quite challenging and very significant. 

In our previous study, ionic liquids were revealed to show a good selective separation of tea polyphenols and caffeine from tea leaves [24]. The adsorption capacity reached 162 mg/g. The adsorption active sites of the ILs are likely partially shielded after immobilization, which made it difficult to achieve a great adsorption capacity. ILs’ polymerization can replace the immobilization and ensure a high availability of active adsorption sites in the adsorption process. 

In this work, a series of imidazole-type bicationic amino acid-based ILs ((ViIm) _2_C_n_ (L-Pro)_2_; n = 3–6) were synthesized, and then new ILs polymers were prepared with the ILs and N,N′-methylene diacrylamide (MBA). In addition to the characterization of the polymers, their adsorption capacities and selectivities for TPs were tested. 

## 2. Experimental Section

### 2.1. Materials and Reagents

N-vinyl imidazole (>98.0%), L-proline and N,N-methylene bisacrylamide were purchased from Titan Scientific Co., Ltd. (Shanghai, China). 1-bromopropane (>98.0%), 1-bromobutane, 1-bromopentane, 1-bromohexane, 1,3-dibromopropane, 1,4-dibromobutane, 1,5-dibromopentane, 1,6-dibromohexane, strong alkalinityanion-exchange resin, sodium acetate, potassium peroxodisulfate, concentrated hydrochloric acid (36%,), ethyl alcohol and methyl alcohol were supplied by Kelong Chemical Co., Ltd. (Chengdu, China). Sodium citrate, sodium chloride, ammonium sulfate, theophylline and tea polyphenol were obtained from Aladdin Biochemical Technology Co., Ltd. (Shanghai, China). All reagents were AR grade with 99% purity, except indicated otherwise. 

### 2.2. Apparatus

Element analysis data were obtained from an EA3000 elemental analyzer (EuroVector Instruments and Software, Milan, Italy). An Infrared L1600300 Spectroscopy (PerkinElmer, Fremont, CA, USA) was used to record Fourier transform infrared spectra (FT-IR) in the range of 4000–400 cm^−1^ with KBr pellets. The morphology was observed with JSM-7001F scanning electron microscopy (JEOL Co., Ltd., Tokyo, Japan). The thermogravimetric analysis (TGA) was performed on Microcomputer differential thermobalance HTG (Hengjiu scientific instrument, Beijing, China) with a heating rate of 10 °C min^−1^ from 30 to 800 °C under nitrogen. The particle size distribution was measured by a JL-1198 laser particle size analyzer equipped with a nonuniform cross arranged three-dimensional sector matrix detector in the range of 0.1–600 μm and an He-Ne gas laser (Run Technology Co. Ltd., Henan, China). The total content of tea polyphenols was determined by a UV-2800 spectrometer (Hengping scientific instrument, Shanghai, China). The quantitative analysis was performed on a high-performance liquid chromatographic system equipped with an LC-20AT pump, an SPD-M20A photodiode array detector and a Class-VP workstation (Shimadzu, Kyoto, Japan). The analytical column was a Symmetry C18 column (4.6 × 250 mm, 5 µm, Waters, MA, USA). The analytical conditions for the actual extracts were as follows: the mobile phase consisted of methanol-water-methanoic acid (20:79.4:0.6, *v/v/v*); the flow rate was 1 mL min^−1^; the column temperature was maintained at 25 °C; the detection wavelength was set at 278 nm, and a 10-µL aliquot of sample solution was injected and analyzed.

### 2.3. Synthesis of Imidazole Ionic Liquid Polymers

#### 2.3.1. Synthesis of ILs monomer ViImCn-L-Pro and (ViIm)2Cn(L-Pro)2

**Figure 1:**
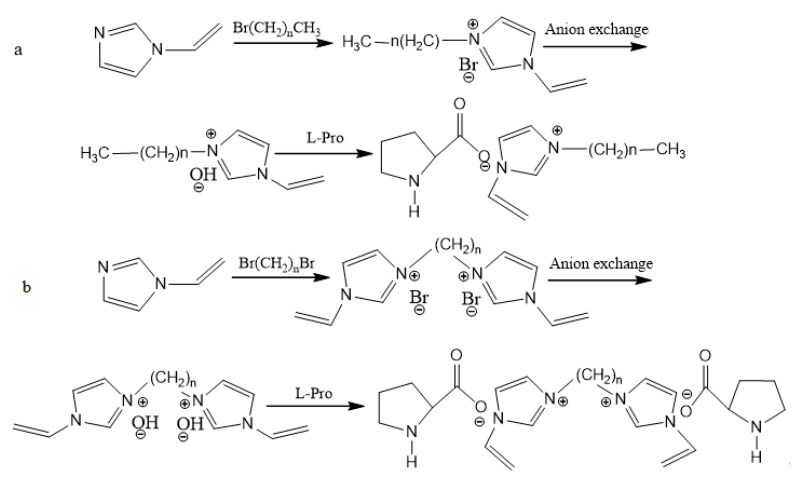
The synthesis of (**a**) ViImCn-L-Pro and (**b**) (ViIm)_2_C_n_(L-Pro)_2_.

The synthetic routes of (ViIm)2Cn(L-Pro)2 are shown in Figure 1. Br(CH_2_)_n_Br and N-Vinylimidazole in a molar ratio of 1:2 were mixed in an appropriate amount of solvent acetonitrile. The mixture was kept at reflux at 82 °C while stirring for 24 h, and then light yellow liquid was obtained. After the solvent was removed, the intermediate product was washed with ethylacetate and acetone in a solid-liquid ratio of 1:10 1–2 times in sequence and was then dried under vacuum for 24 h. An aqueous solution of (ViIm)_2_C_n_(Br)_2_ (n = 3, 4, 5, 6) was made to prepare (ViIm)_2_C_n_OH, with an exchange reaction in styrene 201 × 7 strongly basic anion exchange resin. HPLC was used for monitoring the progress. In the last step, slightly excessive L-proline was added into (ViIm)_2_C_n_OH solution and stirred at room temperature for 24 h. After the reaction, the solvent was removed, and a burgundy viscous oily substance, the target product of ionic liquid (ViIm)_2_C_n_(L-Pro)_2_, was obtained. The product was placed in a low-temperature dry environment for later use. 

The synthesis methods of ViImC_n_-L-Pro were similar to those of (ViIm)_2_C_n_(L-Pro)_2_. The synthesis routes are shown in Figure 1. The structural characterization of the ionic liquid is shown in the Supplementary Materials.

#### 2.3.2. Synthesis of Imidazole Ionic Liquid Copolymers

**Figure 2:**
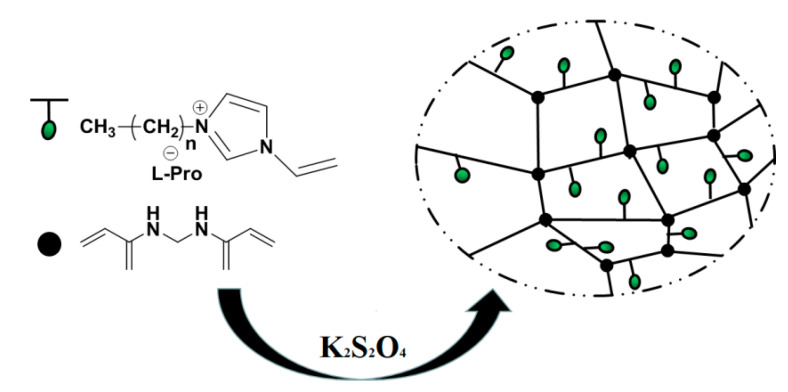
Synthesis routes of imidazole IL copolymers.

ILs as monomers were directly polymerized with cross-linking agent MBA to form polymer. Different molar ratios (0:1, 0.25:1, 0.5:1, 0.6:1, 0.75:1, 1:1) of (ViIm)_2_C_n_(L-Pro)_2_ (n = 3, 4, 5, 6) to MBA were investigated to prepare poly(ionic liquid)s. The six polymers were labeled Pol 0, Pol a, Pol b, Pol c, Pol d, Pol e. The dosage of K_2_S_2_O_4_ was 3.5% (the ratio of the initiator addition to the total mass of the reaction raw materials) as the initiator. The solvent (water, methanol, ethanol, etc.) was ultrasonically vibrated to uniformly disperse the initiator. Inert gas N_2_ was added to exhaust oxygen to carry out the reaction at 50 °C. After the reaction, it was washed twice with pure water and dried for 48 h. The polymerization route is shown in Figure 2.

### 2.4. Structural Characterization of Ionic Liquid Polymer

The poly(ionic liquid)s were characterized with an infrared spectrometer (FT-IR), thermogravimetric analysis (TG), elemental analysis (EA), scanning electron microscope (SEM) and particle size distribution. The infrared spectrum (FT-IR) was measured by transmission method (KBr tableting) on a Perkin Elmer Fourier infrared spectrometer. The thermogravimetric analyzer (TG) was measured by using a microcomputer differential thermal balance. The elemental analysis (EA) was detected by using a EURO EA3000 type element analyzer. The determined elements included C, H, N and O. The content of IL in the polymer was calculated by the content of N, O, and a scanning electron microscope (SEM) was used to observe the surface morphology of the materials. The particle size distribution was measured on a JL-1198 nanolaser particle size analyzer.

### 2.5. Determination of Polymers’ Yield and Mass Ratio of Ionic Liquids in Polymers

In this experiment, a certain amount ViImC_3_-L-Pro was employed as the polymerization monomer, which was directly polymerized with cross-linking agent MBA to form polymer. K_2_S_2_O_4_ was selected as the initiator and accounted for 3% of the total mass of the reaction raw materials. The solvent was water. When this reaction was finished, the polymer was dried for a certain time and weighed. Elemental analysis was used to analyze the content of N, O and to calculate the mass ratio of ILs. The yield was calculated according to the mass of the initial reactants and product. These equations were (1) and (2):

(1) $$\mathrm{mass}\;\mathrm{ratio}=15.75\times \left(1.14P_{N}-P_{O}\right)$$

(2) $$\mathrm{yield}=\frac{m_{\mathrm{polymer}}}{m_{\mathrm{MBA}}+m_{\mathrm{ILs}}}$$

P_N_ is the content of N in the polymer. P_O_ is the content of O in the polymer. m_Polymers_(g) is the mass of the polymer. m_MBA_(g) is the quality of the initial response of the MBA. m_Ils_(g) is the quality of the initial response of the Ils. 

### 2.6. Optimization of Polymer Synthesis Conditions 

#### 2.6.1. Influence of the Ratio of Ionic Liquid and Crosslinking Agent MBA on Adsorption

0.6 g of cross-linking agent (MBA) was added in 30 mL distilled water to form a solution. Then, a certain amount of IL (ViImC_3_-L-Pro) was dissolved in the solution with ultrasound assistance and was left standing for three hours. An appropriate amount of initiator K_2_S_2_O_4_ was dissolved in the solution with ultrasound assistance. Nitrogen gas was introduced for 15 min to remove oxygen, the solution was placed at 50 °C for 6 h, and a light yellow polymer was obtained. It was washed twice with pure water and dried under vacuum for 48 h. A polymer of 200–350 mesh was prepared by grinding and sieving it for the adsorption test.

#### 2.6.2. Influence of Solvents on Adsorption

Water, methanol and ethanol were used, respectively, as a polar solvent to investigate their influence. K_2_S_2_O_4_ was used as the initiator when water was used as a solvent. However, AIBN was used as the initiator when methanol or ethanol were used as a solvent, due to the lower solubility of K_2_S_2_O_4_ in methanol or ethanol. The polymerization was carried out at a ratio of n(IL):n(MBA) = 1:3.

#### 2.6.3. Influence of Ionic Liquids on Adsorption

Under the ratio of n(IL):n(MBA) = 1:3, (ViIm)_2_Cn(L-Pro)_2_(n = 3, 4, 5, 6) and ViImCn-L-Pro(n = 3, 4, 5, 6) were taken as monomers, respectively, in the case of a water solvent and a K_2_S_2_O_4_ initiator in order to synthesize the polymers. 

#### 2.6.4. Influence of Initiator Amount on Adsorption

(ViIm)_2_C_6_(L-Pro)_2_ was used as a monomer. Under the optimal ratio of n(IL):n(MBA), a total mass of the initiator K_2_S_2_O_4_ of 2.5%, 3.5%, 4.5%, 6.0%, 9.0% and 12% was selected, respectively, to synthesize the polymer in the water solvent. 

### 2.7. Adsorption Test of TPs

#### 2.7.1. Establishment of Determination Method for TPs

The total TP content in the adsorption experiment was analyzed by using the area normalization method by comparing the spectra with the standard tea polyphenols and theophylline that were purchased.

#### 2.7.2. Adsorption Performance Test

40.0 mg polymer (mesh size 200–350) was mixed with a 20.0 mL solution of 1 mg/mL TP concentration, and the mixture was placed in a vibrator (200 rpm) at a certain temperature for the TPs’ adsorption. The adsorption conditions were investigated under the following conditions: the solid-liquid ratio was 60:20; the adsorption temperature was 25 °C; the adsorption time was 6 h; and the initial concentration range was between 0.5–3.5 mg/mL. After 360 min, 1.00 mL of the supernatant sample was taken and analyzed at 273 nm by HPLC to determine each compound’s content with an area normalization. Then, the TPs’ adsorption capacity *q*_e_(mg/g) and the adsorption selectivity *S* of the adsorbent could be calculated according to Equations (3) and (4):

*q*_e_ = mass (mg) of absorbed TPs/mass (g) of absorbent (3)

*S* = mass (mg) of absorbed TPs/mass (mg) of absorbed theophyline (4)

## 3. Results and Discussion

### 3.1. Characterization of Ionic Liquid Polymers

#### 3.1.1. Infrared Spectroscopic Analysis

**Figure 3:**
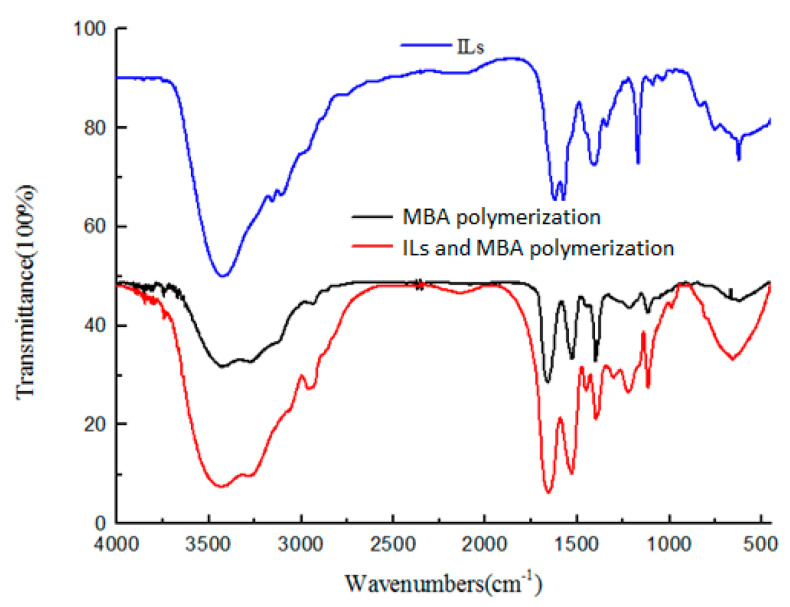
The IR spectrum of IL, MBA and Pol c.

The infrared spectrum characterization results of IL (ViImC_3_-L-Pro), Pol 0 (MBA polymer only) and Pol c ((ViImC_3_-L-Pro):MBA = 3:5) are shown in Figure 3. The main characteristic peaks of Pol 0 are the bending vibration absorption peak of C=O at 1651 cm^−1^ and the strong absorption peak of the N-H bending vibration at 1528 cm^−1^ (unique to the N-H anti conformation) [25]. Similarly, corresponding absorption peaks could also be found at the corresponding positions in Pol c, which meant that MBA components participated in the formation of Pol c. The absorption peak of Pol c at 3429 cm^−1^ was significantly enhanced when comparing the infrared signals of Pol 0 and ILs. It was possible that the water absorption of amino acid-based ILs itself was particularly strong and that the water absorption peak of Pol c was therefore stronger. At the same time, the absorption peaks of Pol c at 1385 cm^−1^, 1225 cm^−1^, 1176 cm^−1^ and 760 cm^−1^ were also obviously enhanced, which could be due to the addition of the IL component [26]. The significant difference between the infrared spectra of Pol c and Pol 0 was that a new peak appeared at 1459 cm^−1^, which was the absorption peak of the imidazole ring in IL. According to the above spectrum signal characteristics, it could be initially proven that IL took part in the polymerization reaction and that the expected copolymer was formed.

#### 3.1.2. Elemental Analysis

Amino acid-based ILs have a stronger polarity. No solid polymer was formed in the reaction solution with only the initiator. After MBA was added, the polymerization reaction could take place, and so MBA was an important component to realize the formation of the polymer network structure. In this experiment, ViImC_3_-L-Pro was employed as the polymerization monomer, and K_2_S_2_O_4_ was selected as the initiator and accounted for 3% of the total mass of the reaction raw materials. The elemental analysis results and the molar ratio of the ILs and crosslinking agent are listed in Table 1 and Table 2. After the polymerization reaction was finished, the obtained polymer molecules contained IL and MBA components synchronously. Elemental analysis was used to measure the content changes of C, H, N and O elements in the polymers. Based on the percentage of N and O in the ILs and MBA, the content of ionic liquid in the polymer could be calculated. The yield could be obtained by changing the mass before and after the reaction. 

The contents of N in MBA and ViProIm+-L-Pro- molecules were 18.2% and 16.7%, respectively, and the contents of O in MBA and ViProIm+-L-Pro- molecules were 20.8% and 12.7%, respectively. After the polymerization reaction was finished, the obtained polymer molecules contained IL and MBA components synchronously. Elemental analysis was used to measure the content changes of C, H, N and O elements in the polymer molecules, which was used to calculate the mass fraction of IL in the polymer molecules. With the increase of the proportion of ionic liquid IL, its mass fraction in the polymer could reach up to more than 30%. On the contrary, the polymer yield decreased with the increase of the proportion of IL. When the ratio of n (IL): n (MBA) was more than 0.75:1, the solid polymer could not be obtained, even if the amount of initiator was increased. 

#### 3.1.3. TGA-DTG Analysis

**Figure 4:**
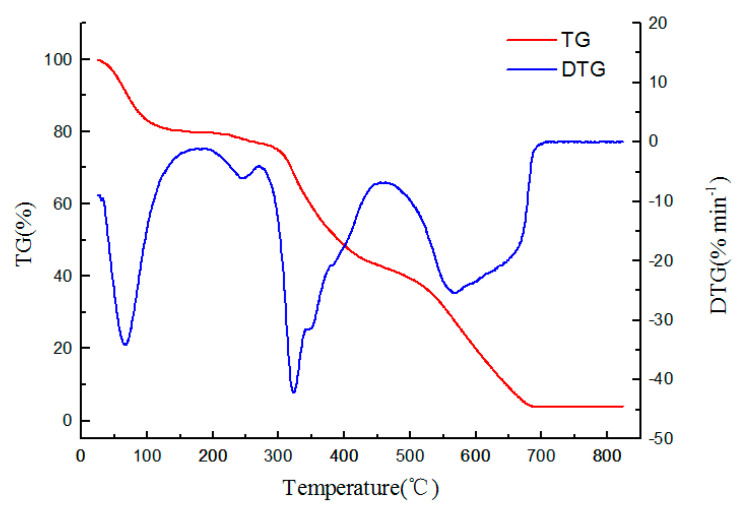
TGA and DTG analyses of the IL polymer.

The product Pol c, a reaction ratio of IL: MBA of 0.6:1, was taken as an example. TG and DTG analyses were conducted to study the thermal performance of changes in the polymer mass with the temperature. The results are shown in Figure 4. 

It can be seen from the DTG curve that a mass loss below 100 °C was caused by water evaporation and accounted for about 20% of the total weight, which may be due to an incomplete drying of the polymer after synthesis. When the temperature was higher than 310 °C, the polymer began to lose weight rapidly. 

It could be seen from Figure 4 that three large weight loss peaks appeared. The part under 100 °C was the reason for the loss of residual water. In addition, there was a particularly small weight loss peak at 245 °C. The weight loss peak was not completely separated from the weight loss peak at 310 °C. It could be inferred that the polymer was decomposed in three steps under the heating conditions. Moreover, the critical temperature of the two parts was relatively close around the falloff. From 200 °C–250 °C, the amino group in the polymer began to decompose and fall off at first. At the same time, a small amount of quaternary ammonium cation degraded to form volatile components [27]. When the temperature reached 280 °C, a large weight loss peak appeared, and the degradation speed was faster. A large amount of MBA components and quaternary ammonium cation began to degrade [28]. When the temperature was over 480 °C, the polymer carbon skeleton started to collapse. A large weight loss peak appeared at around 540 °C. Then, the weight loss rate started to slow down. After 680 °C, the weight loss reached a constant. There was very little remaining substance on the thermobalance, which might have been indicative of residual ash. The figure shows that the polymer had a great thermal stability below 60 °C.

#### 3.1.4. Analysis of SEM Surface Structure

**Figure 5:**
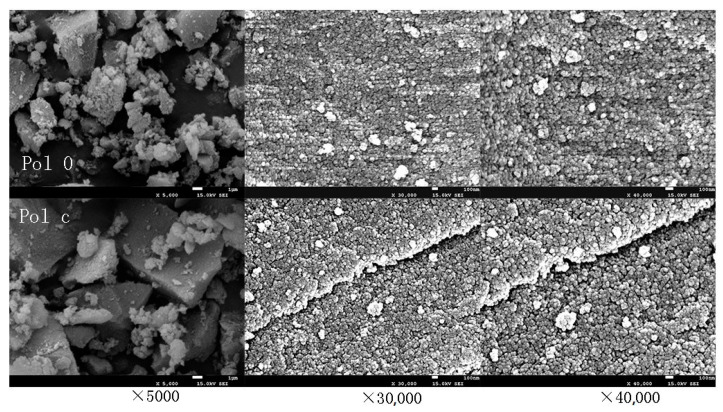
The SEM characterization of Pol 0 and Pol c.

Figure 5 shows the SEM diagram of the polymer surface structure. By comparing Pol 0 and Pol c, it was found that the surface of the synthesized polymer was significantly rougher and more porous after the addition of IL liquid. When ionic liquid was added to the polymer, the surface roughness was caused by the different structure of each connecting point of the polymer. It was found that the polymers were composed of nanoscale particles. Therefore, it could be concluded that the ionic liquid polymer had a rough and porous surface and was composed of numerous nanoscale particles.

#### 3.1.5. Particle size Distribution

**Figure 6:**
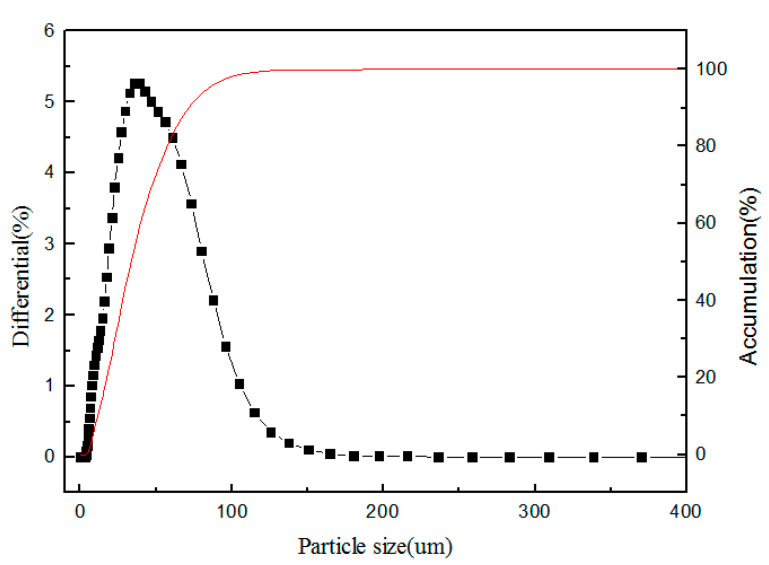
The particle distribution of polymers.

The polymer in Figure 6 was a pol c polymer. It was found that the particle size distribution of the polymer was not uniform and that the main particle size distribution was 40 μm to 60 μm in Figure 6. This was mainly because the nanoparticle polymer continuously agglomerated to form a macroscopic polymer in the synthesis process. SEM research had shown that the component unit of the ionic liquid polymer was a nanometer particle, so one could see that sufficient agitation was needed in the adsorption process to break the agglomeration effect as much as possible. The smaller the particle size was, the larger the specific surface area was, which was favorable for adsorption.

### 3.2. Influence of Synthesis Conditions of Ionic Liquid Polymer on Adsorption Effect

#### 3.2.1. Influence of the Ratio of ILs and MBA on Adsorption

**Figure 7:**
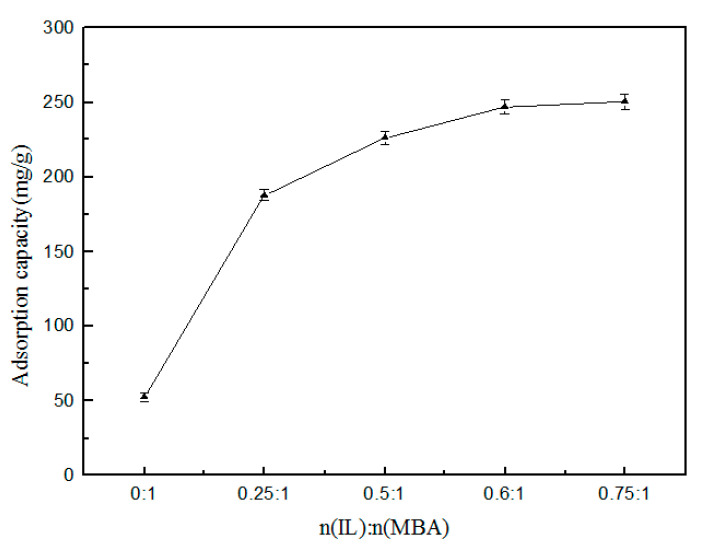
Effect of the amount of n(IL)/n(MBA).

The results of the influence of the ratio of IL and MBA in synthetic raw materials on the adsorption effect of polymer are shown in Figure 7. It could be seen from Figure 7 that the polymer formed by the polymerization of the cross-linking agent only had a poor adsorption effect on TPs, at only 60 mg/g. The reason for this was that the polymer formed by MBA had a lower affinity for TPs, which resulted in a poor adsorption effect. When IL and MBA were added for polymerization, the adsorption performance of the obtained polymer was greatly improved. With the proportion of IL and MBA in the synthetic formula gradually increased, the adsorption capacity of the synthesized gel on TPs increased rapidly at first, before gradually stabilizing. When the ratio of IL to MBA continued to increase, the polymerization could not proceed. The reason for this was that the amount of cross-linking agent MBA was insufficient. Moreover, it was found that, with the increase of the ratio of IL to MBA, the yield of the polymer was also lower during the experiment. Considering the economic cost and adsorption effect, n(IL):n(MBA) = 0.6:1 was selected as the essential condition for the next experiment.

#### 3.2.2. Influence of Solvent Types on Adsorption

The effect of the solvent type on the adsorption of TPs by the synthesized polymer is shown in Table 3. It was found that the differences of adsorption were not significant when three different solvents were used. Among them, when methanol was used as the solvent, the synthesized polymer had the best adsorption effect on TPs. When water was used as the solvent, the polymer had the second best adsorption effect. In view of green synthesis, water was suggested as a solvent, rather than methanol, although the result from methanol was somewhat better than that from water. Furthermore, if possible residuals of the solvent were to be considered, they might show a potential risk in food and pharmaceutical processes, and a water solvent would therefore be preferential.

#### 3.2.3. Influence of Ionic Liquids on Adsorption

**Figure 8:**
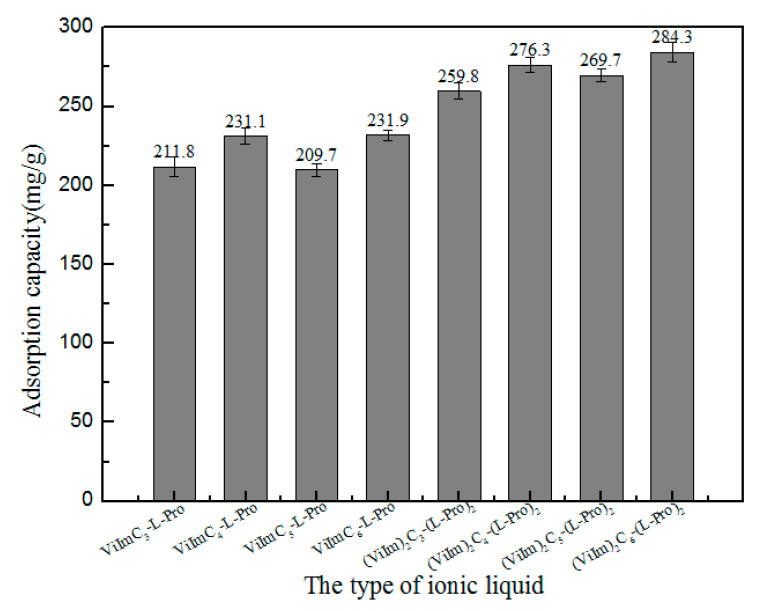
The types of ILs.

The adsorption of TPs by polymers formed by the polymerization of IL and MBA with different kinds of ILsis shown in Figure 8. It could be seen from Figure 8 that, when all adsorption conditions were the same, the adsorption effect of the polymer formed by double positive ILs on TPs was better than that of the polymer formed by single positive ILs. The reason for this was that when n(IL):n(MBA) (dicationic ILs were half of the ratio of single positive ILs) was not changed, dicationic IL had two sites that could polymerize with the crosslinking agent, which made it easier for the chain structure to form a network polymer and convert into molecules with a complex spatial structure. The situation was beneficial to the adsorption of TPs. In addition, with the increase of the carbon chain, the unit adsorption capacity of the polymer also showed an increasing trend. Finally, (Vilm)_2_C_6_-(L-Pro)_2_ was selected for further research.

#### 3.2.4. Influence of Amount of Initiator on Adsorption

**Figure 9:**
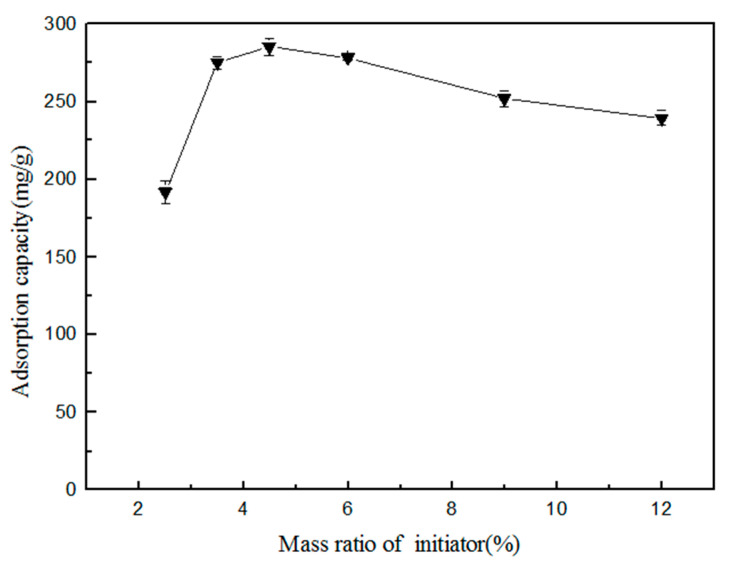
The mass ratio of the initiator.

Because the addition of the initiator K_2_S_2_O_4_ was less than two percent of the total mass of the reaction raw materials, the polymerization could not occur and the polymer could not be formed; therefore, the addition of K_2_S_2_O_4_ in the experiment was investigated by starting at 2.5 percent. The effect of the added amount of initiator K_2_S_2_O_4_ in the formula on the adsorption amount of the formed polymer on TPs is shown in Figure 9.

It could be seen from Figure 9 that, when the added amount of the initiator K_2_S_2_O_4_ gradually increased, the adsorption amount of the synthesized polymer for TPs showed an increasing trend at first, before decreasing. Additionally, the added amount of K_2_S_2_O_4_ was best around 4.5%. The formed polymer had the maximum adsorption amount for TPs. In the synthetic polymers, both ILs and MBA had certain adsorption effect on TPs, but the adsorption effect of MBA was very small. The main effect of the MBA component was to form a polymer network structure to provide support. When the amount of the initiator K_2_S_2_O_4_ was less than two percent, its ability to release free radicals was limited, and polymerization could not occur. When the amount of the initiator K_2_S_2_O_4_ increased from 2.5% to 4.5%, the free radicals released by the initiator K_2_S_2_O_4_ increased, which increased the probability of ILs and MBA to obtain free radicals. The polymerization reaction occurred, and the polymer was formed. When the amount of the initiator K_2_S_2_O_4_ was higher than 4.5%, this was still not conducive to the full polymerization of the functional monomer and crosslinking agent, although the polymerization reaction could be completed within a very short time. Eventually, the polymerization between the monomer or crosslinking agent affected the final adsorption effect.

#### 3.2.5. The Performances of Adsorption and Reuse

**Figure 10:**
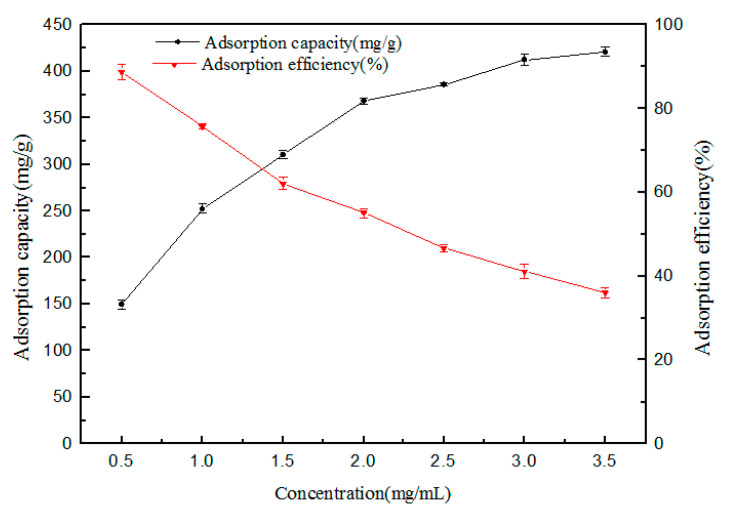
Effect of the initial concentration on the extraction efficiency.

The results are shown in Figure 10. With the increase of the initial concentration of tea polyphenols solution, the unit adsorption capacity of the adsorbent increased and, on the contrary, the adsorption rate decreased. When the initial concentration of tea polyphenols increased from 0.5 mg/mL to 3.5 mg/mL, their adsorption capacity increased from 149 mg/g to 420 mg/g, and their adsorption efficiency decreased from 88% to 36%.This was mainly because when the concentration was relatively low, the adsorption sites of the adsorbent were abundant and the substrates were few, which could make most of the tea polyphenols be adsorbed. However, the total amount of tea polyphenols was limited, so the adsorption amount was low. With the increase of the initial concentration, the total amount of tea polyphenols increased significantly, the adsorption sites of the adsorbent were gradually occupied, the adsorption amount increased gradually, and the adsorption rate decreased continuously.

The adsorption performance of TPs depended mainly on both the properties of the ionic liquid polymers and the adsorption conditions. The primary adsorption conditions, including the time, solid-liquid ratio, solution pH and temperature, were investigated. A typical adsorption performance obtained with the Multiple factors was 521 mg/g (synthesis conditions: solvent methanol, initiator K_2_S_2_O_8_, ILs (ViIm)_2_C_3_(L-Pro)_2_, n (ILs): n (MBA) = 0.3:1; adsorption conditions: solid-liquid ratio 6:2 mg:mL, 45 °C, 6 h, pH 6.0), which was greatly improved [29].

A mixed solution of TPs and theophylline was used to verify the selectivity, and the results are shown in Table 4. The extracting solution from raw material tea is usually rich in both TPs and a certain amount of theophylline, which are very similar in structure. Thus, their separation is necessary and more difficult. After adsorption, the TPs’ content in the solution decreased remarkably, while the theophylline content was almost unchanged. Therefore, the selectivity was calculated at 31.85, according to Equation (4). This indicates that the adsorption material had a very high selectivity to TPs when compared to theophylline.

The absorbed TPs on the polymer could be desorbed satisfactorily by using a 2% hydrochloric acid methanol solution as eluent. Table 5 shows the variation of the adsorption capacity after the desorption. The capacity decreased gradually over four runs. The decrease could be affected by multiple factors, such as the eluent’s property, the conditions of reuses, and, particularly, its performance of adsorption. Further studies should be conducted on the performance stability and recovery conditions for practical industrial applications.

#### 3.2.6. FTIR study on Adsorption Process

**Figure 11:**
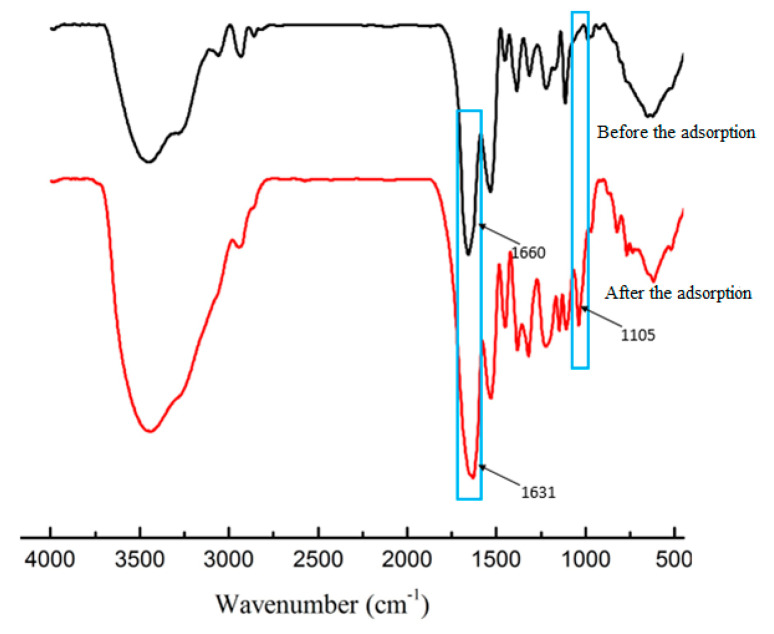
IR spectrum of the polymer before and after adsorption.

The infrared spectrum of the polymer before and after adsorption is shown in Figure 11. It could be seen that the characteristic absorption peak of the imidazole ring v (C=N) and v (C=C) decreased from 1660 cm^−1^ to 1631 cm^−1^ before adsorption, which indicated that the imidazole ring of the polymer and TP was formed as a formed π-π conjugate effect, with the absorption peaks showing a red-shift. In addition, it was found, by comparing the polymer adsorption before and after, that a stronger absorption peak was added at 1105 cm^−1^. This was the characteristic absorption peak of v (C-O) of TPs [30].

## 4. Conclusions

New polymer materials can be prepared by the polymerization of imidazole-type biscationic amino acid-based ILs and MBA. A typical adsorption performance obtained with this comprehensive investigation was 521 mg/g (synthesis conditions: solvent water, initiator K_2_S_2_O_8_, ILs (ViIm)_2_C_6_(L-Pro)_2_, n (ILs): n (MBA) = 0.3:1; adsorption conditions: solid-liquid ratio 6:2 mg:mL, 45 °C, 6 h, pH 6.0), which was significantly higher than for other adsorption media.

In summary, ionic liquid polymers can be considered as a promising material for the separation of natural products, demonstrating an ideal adsorption capacity and good selectivity. They can be used in the separation of TPs from mixtures or actual samples; meanwhile, they provide a useful reference for the separation of similar components.

## Figures and Tables

**Table 1:** The elemental analysis results.

Polymer Monomer	C	H	O	N
MBA	54.5%	6.5%	20.8%	18.2%
ViProIm+-L-Pro-	66.6%	4%	12.7%	16.7%
Pol c	59.9%	5.2%	17.7%	17.2%

**Table 2:** The recipe of IL Pols and their EA analysis results.

ILs Polymer	n (IL):n (MBA)	Mass Ration of IL (%)	Yield (%)
Pol 0	0:1	0	95.1
Pol a	0.25:1	15.9	82.3
Pol b	0.5:1	28.6	55.7
Pol c	0.6:1	29.9	50.9
Pol d	0.75:1	31.02	28.6
Pol e	1:1	-	-

**Table 3:** Effect of the types of solvents.

Solvent	Adsorption Capacity (mg/g)	Change in Capacity (%)
Ethanol	231.5	0
Water	239.9	3.6
Methanol	283.8	22.6

**Table 4:** Compounds in the tea solution before and after adsorption.

Adsorption	Compound	Each Compound (%)	Concentration of TPs or Theophylline in Solution (mg/mL)
Before adsorption	ECGC	46.4	0.40
	EC	11.6	
	ECG	4.3	
	Theophylline	37.7	0.075
After adsorption	ECGC	7.5	0.15
	EC	15	
	ECG	10	
	Theophylline	67.5	0.068

**Table 5:** Variations of the adsorption capacity with reuses.

Run Times	1	2	3	4
adsorption capacity(mg/g)	521	515	500	476
Variation%	0	−1.2	−4.0	−8.6

## Supplementary Materials

The following are available online at https://www.mdpi.com/2073-4360/12/10/2171/s1.
