# Supplementary material for: Synthesis of a New Imidazole Amino Acid Ionic Liquid Polymer and Selective Adsorption Performance for Tea Polyphenols
Source: Polymers (Basel). 2020 Sep 23;12(10):2171. doi: 10.3390/polym12102171 (PMC7598198; doi:10.3390/polym12102171)
Supplement: Supplementary file 1 [file polymers-12-02171-s001.pdf]

Nuclear magnetic hydrogen spectrum of vinyl imidazole

(a): $[ViIm]_2C_3[L\text{-}pro]_2$; (b): $[ViIm]_2C_4[L\text{-}pro]_2$; (c): $[ViIm]_2C_5[L\text{-}pro]_2$; (d):$[ViIm]_2C_6[L\text{-}pro]_2$; (e):$[ViIm]_2C_3[L\text{-}pro]_2$; (f): $[ViIm]_2C_4[L\text{-}pro]_2$; (g): $[ViIm]_2C_5[L\text{-}pro]_2$;(h): $[ViIm]_2C_6[L\text{-}pro]_2$

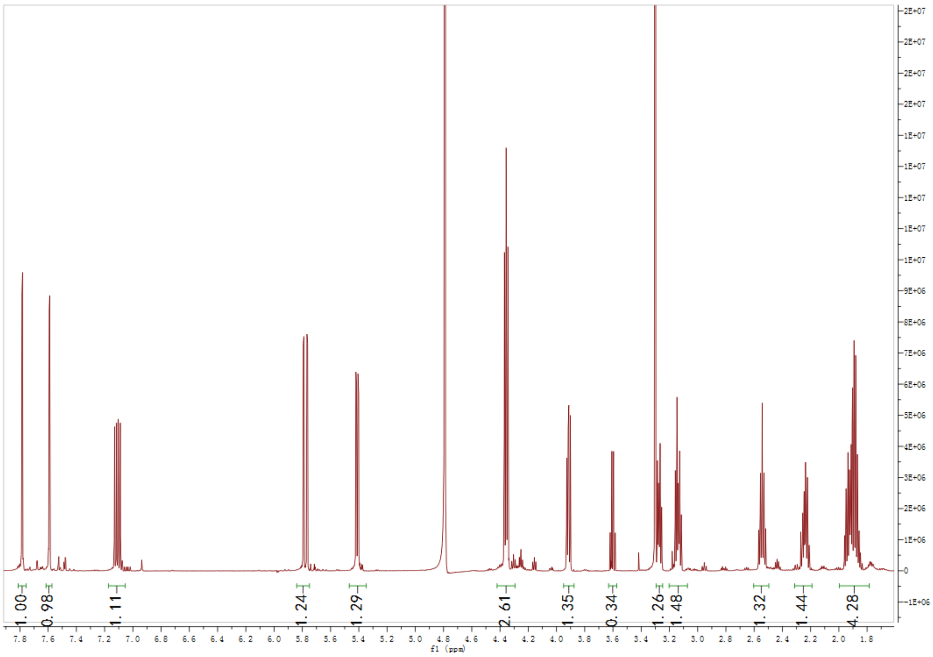

$[ViIm]_2C_3[L\text{-}pro]_2$: ^{1}H NMR (400 MHz, D_2O) δ7.79 (d, 2H),7.59 (d, 2H),7.13-7.09(m,2H),5.79-5.76 (m, 2H),5.42-5.40 (m, 2H),4.37-4.34 (m, 4H),3.93-3.90 (m, 2H), 3.29-3.27 (m, 2H),3.15-3.13 (m, 2H),2.56-2.53 (m, 2H),2.25-2.23 (m, 2H),2.22-1.88 (m, 6H).

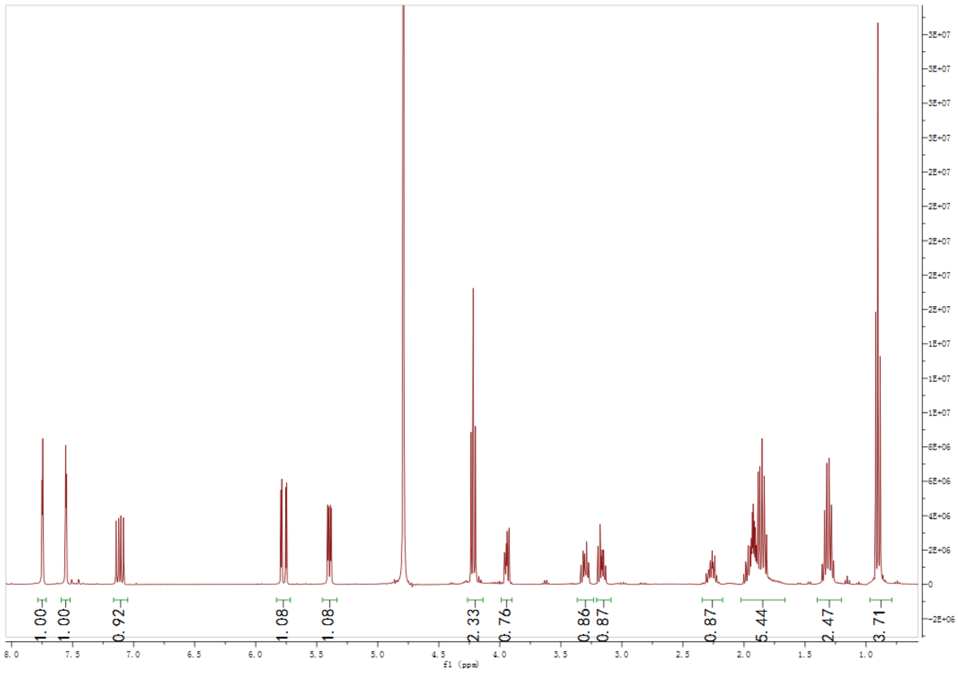

$[ViIm]_2C_4[L\text{-}pro]_2$: ^{1}H NMR (400 MHz, D_2O) δ7.75 (d, 2H),7.56 (d, 2H),7.14-7.08(m,2H),5.79-5.75 (m, 2H),5.41-5.38 (m, 2H),4.24-4.20 (m,

4H),3.95-3.90 (m, 2H), 3.29-3.27 (m, 2H),3.18-3.15 (m, 2H),2.28-2.26 (m, 2H),1.93-1.90 (m, 4H),1.32-1.27 (m,4H), 0.92-0.88 (m,2H).

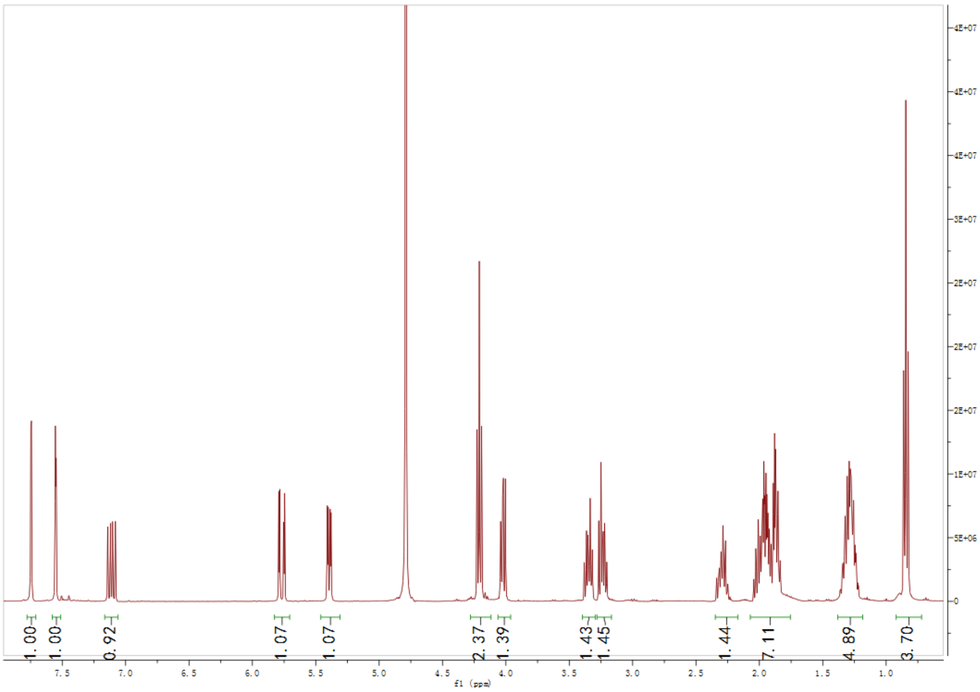

[ViIm]$_2$C$_5$[L-pro]$_2$: ^{1}H NMR (400 MHz, D_2O) δ7.75 (d, 2H),7.55 (d, 2H),7.14-7.08(m,2H),5.79-5.75 (m, 2H),5.41-5.38 (m, 2H),4.24-4.19 (m, 4H),4.03-4.00 (m, 2H), 3.33-3.32 (m, 2H),3.27-3.22 (m, 2H),2.29-2.26 (m, 2H),1.97-1.90 (m, 8H),1.32-1.27 (m,4H), 0.92-0.88 (m,2H).

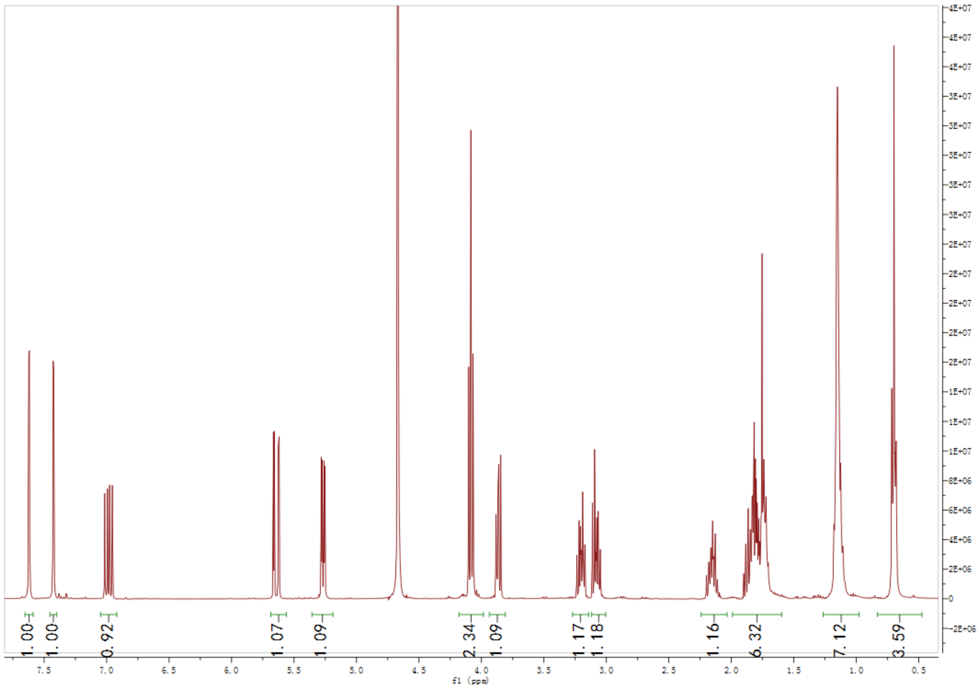

[ViIm]$_2$C$_6$[L-pro]$_2$: ^{1}H NMR (400 MHz, D_2O) δ7.74 (d, 2H),7.55 (d, 2H),7.14-7.07(m,2H),5.79-5.74 (m, 2H),5.40-5.37 (m, 2H),4.22-4.19 (m, 4H),3.99-3.96 (m, 2H), 3.23-3.20 (m, 2H),320-3.19 (m, 2H),2.28-2.25 (m, 2H),1.96-1.93 (m, 8H),1.28-1.23 (m,8H), 0.82-0.80 (m,2H).

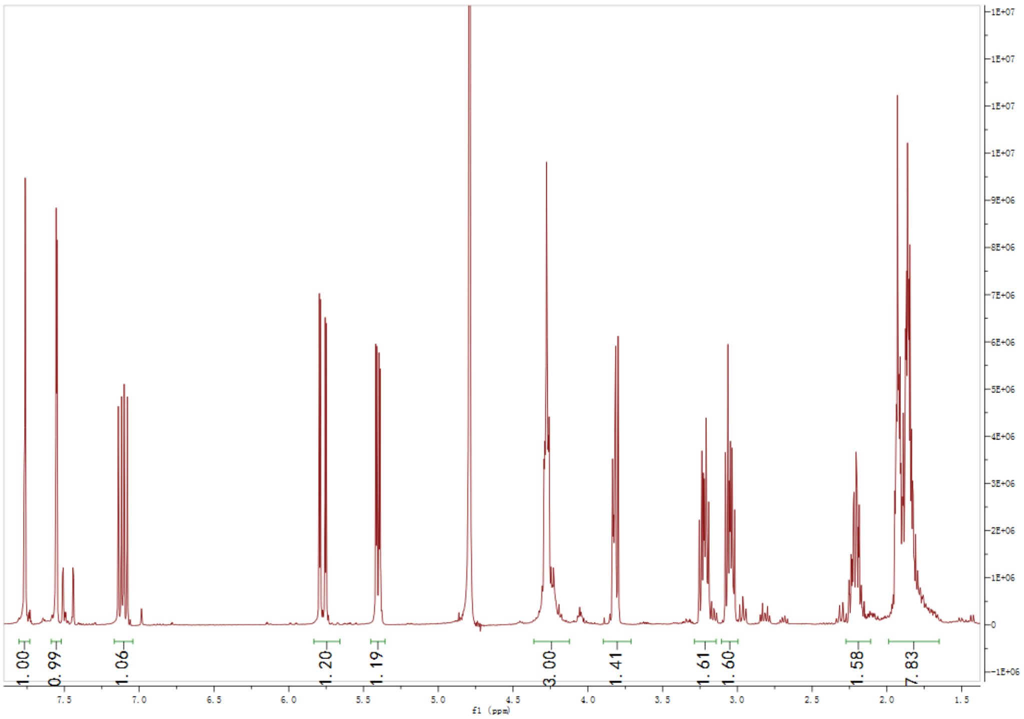

ViImC$_3$-L-pro: ^{1}H NMR (400 MHz, D_2O) δ7.76 (d, 1H),7.55 (d, 1H),7.14-7.08(m,1H),5.80-5.75 (m, 1H),5.42-5.39 (m, 1H),4.27-4.22 (m, 2H),3.83-3.81 (m, 1H), 3.21-3.20 (m, 1H),3.05-3.03 (m, 1H),2.22-2.18 (m, 1H),1.88-1.81 (m, 4H)。

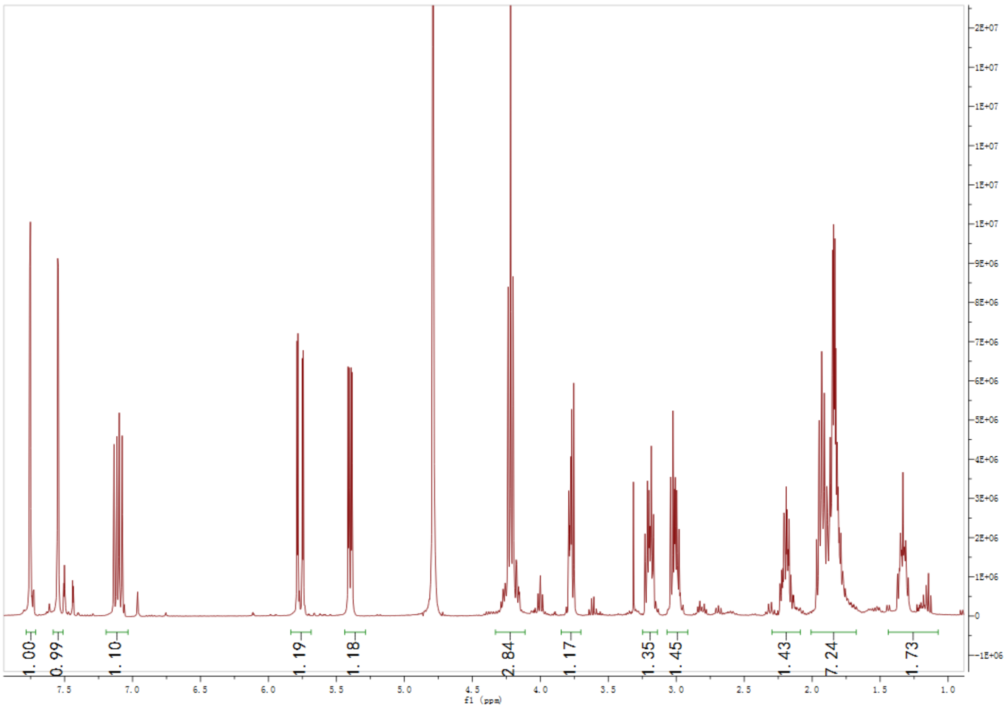

ViImC$_4$-L-pro: ^{1}H NMR (400 MHz, D_2O) δ7.75 (d, 1H),7.44 (d, 1H),7.12-7.08(m,1H),5.79-5.73 (m, 1H),5.41-5.39 (m, 1H),4.24-4.20 (m, 2H),3.75-3.61 (m, 1H), 3.32-3.17 (m, 1H),3.02-3.00 (m, 1H),2.21-2.14 (m, 1H),1.88-1.74 (m, 4H)，1.35-1.14 (m, 2H)。

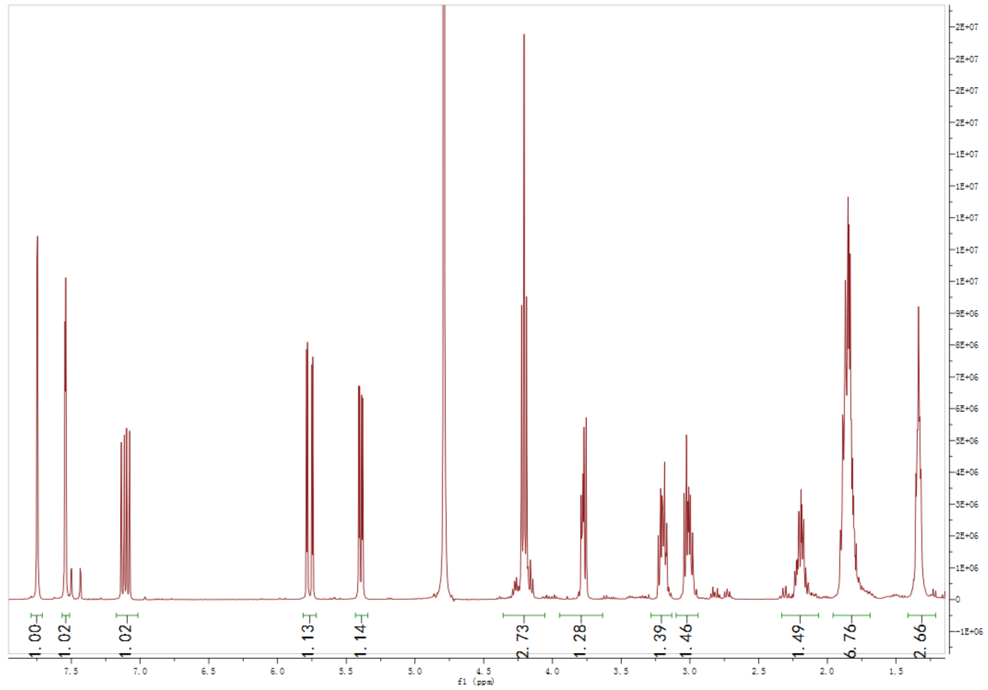

ViImC$_5$-L-pro: 1H NMR (400 MHz, D2O) δ7.75 (d, 1H),7.43 (d, 1H),7.14-7.08(m,1H),5.79-5.74 (m, 1H),5.41-5.38 (m, 1H),4.21-4.18 (m, 2H),3.79-3.76 (m, 1H), 3.19-3.17 (m, 1H),3.03-3.00 (m, 1H),2.21-2.17 (m, 1H),1.86-1.747 (m, 6H)，1.35-1.32 (m, 2H)。

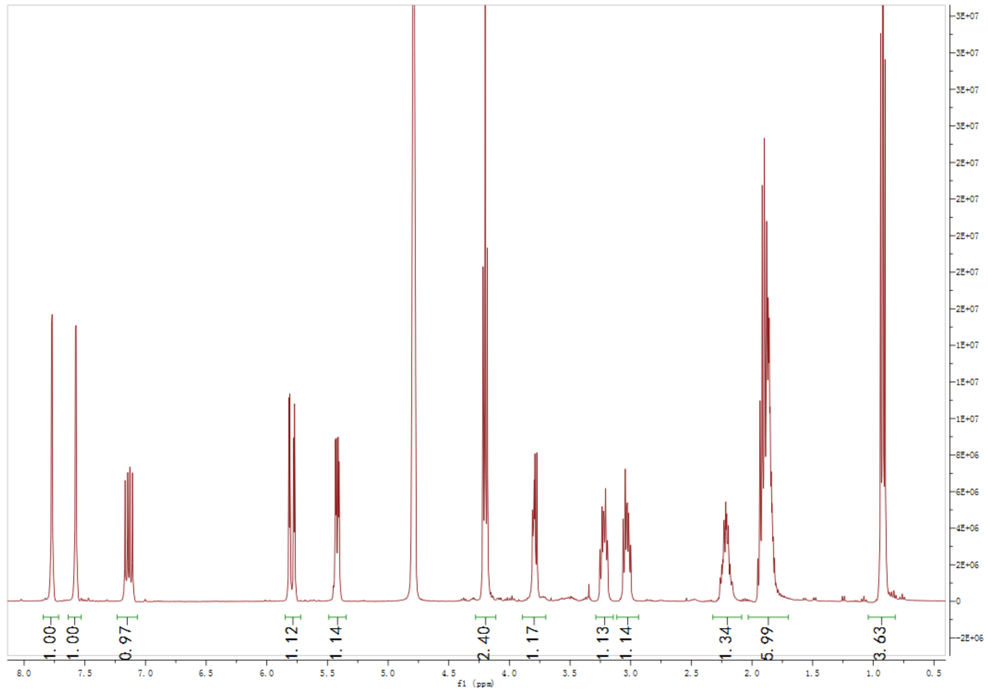

ViImC$_6$-L-pro: ^{1}H NMR (400 MHz, D_2O) δ7.77 (d, 1H),7.58 (d, 1H),7.17-7.11(m,1H),5.82-5.77 (m, 1H),5.43-5.41 (m, 1H),4.22-4.18 (m, 2H),3.81-3.71 (m, 1H), 3.27-3.20 (m, 1H),3.04-3.00 (m, 1H),2.21-2.20 (m, 1H),1.92-1.81 (m, 6H)，0.94-0.90 (m,4H)。
